# Staff perspectives on racial inequities in the neonatal intensive care unit: the REJOICE study

**DOI:** 10.1038/s41372-025-02378-y

**Published:** 2025-08-06

**Authors:** Krystal Austin, Olga Smith, Monica McLemore, Sarah Lewis, Safyer McKenzie-Sampson, Taylor E. Washington, Elizabeth E. Rogers, Kayla L. Karvonen

**Affiliations:** 1 University of Colorado School of Medicine, Aurora, CO USA; 2 Independent Researcher, Antioch, CA USA; 3 Department of Child, Family, and Population Health Nursing, University of Washington, Seattle, WA USA; 4 College of Pharmacy, Touro University, Vallejo, CA USA; 5 Department of Pediatrics, Stanford University, Palo Alto, CA USA; 6 University of Virginia School of Medicine, Charlottesville, VA USA; 7 Department of Pediatrics, University of California San Francisco, San Francisco, CA USA; 8 California Preterm Birth Initiative, San Francisco, CA USA

**Keywords:** Health occupations, Ethics

## Abstract

**Objective:**

To understand staff perspectives on racism experienced by both parents and staff members in the neonatal intensive care unit (NICU).

**Study design:**

Open-ended surveys and semi-structured interviews were conducted with staff at an urban level IV NICU from 2021 to 2022. Themes were generated and refined using thematic analysis. The main outcome constituted participants’ experiences of structural racism.

**Results:**

72 multi-disciplinary and racially and ethnically diverse participants completed the survey and 10 participants were also interviewed. Five major themes were identified: (1) a wide range of denial and recognition of racism existed, (2) workplace culture and relationships both protected against and facilitated racism, (3) staff experienced a lack of workforce diversity and minority tax, and witnessed (4) biased communication and language barriers, and (5) disparate resource allocation.

**Conclusions:**

Similar to other healthcare worker and caregiver reports, NICU staff members also experience and witness interpersonal, institutional, and structural forms of racism.

## Background

Racism is associated with adverse healthcare outcomes, contributing to racial disparities in preterm birth and low-birth weight, quality of care, and postnatal outcomes [1–4]. Studies have shown that structural racism, or systems-level conditions that limit opportunities, resources, power, and the well-being of people based on race/ethnicity, creates this phenomenon [5, 6]. In addition, institutionalized, interpersonal, and internalized forms of racism work together to perpetuate racial inequities [7]. In the neonatal intensive care unit (NICU), structural, institutional, and interpersonal racism has multifaceted manifestations, including but not limited to: the societal inequities that lead to increased rates of preterm birth, disparate application of policies and policing for racially marginalized groups, regionalization and segregation of quality care, homogenous staff demographics, and interpersonal discrimination [8–13].

Much of the literature on racism in the healthcare setting has focused on interpersonal manifestations, and descriptions exist across many disciplines [14]. Similar themes have been described by NICU families who have reported biased provider attitudes that lead to disparate treatment or dismissed concerns [8, 15–21]. Black families have also highlighted the lack of racially congruent providers caring for their infants and fears that their infants might be mistreated as a result [15, 16]. Historic and contemporary scientific literature has shown that patients’ fears of being mistreated by healthcare systems are not unfounded [14, 22–24].

Healthcare providers also experience racism, both vicariously through witnessed patient and family experiences and their own personal experiences [25]. Racially marginalized physicians experience racist microaggressions both from patients and staff, and these are positively correlated with a measure of secondary traumatic stress [26]. Racially marginalized nurses also experienced racism and sexism related stress, emotional labor, and depleted emotional resources [27].

Although previous literature has investigated staff experiences with racism broadly, there are very few studies of multi-disciplinary staff experiences with racism in pediatrics and more specifically, neonatology. Within the broader Racial and Ethnic Justice in Outcomes in Neonatal Intensive Care (REJOICE) study, we sought to identify NICU staff experiences of racism and discrimination within an urban level IV regional NICU. With this analysis, we sought to build upon the existing body of work by elaborating upon the intersections of race, gender, and staff roles.

## Methods

### Settings and participants

Convenience sampling was used to recruit participants from a single urban, level IV NICU in the San Francisco Bay Area, California. Eligibility criteria for participation included all staff employed in the NICU during the study period April 2021–October 2022 who spoke English. Ninety-two staff completed survey data, 72 of whom responded to open-ended questions, and 10 of whom opted-in to participate in one-on-one qualitative interviews. This study was limited to the 72 staff who provided qualitative data through open-ended survey responses and/or an interview.

### Procedures

Staff members were invited via email and in-person recruitment to participate in surveys that included demographic and open-ended questions. Staff members who participated in the survey were also invited to participate in semi-structured interviews on a virtual platform. Interviews were conducted by research assistants who had participated in interview training, and had no prior relationships with the participants. Virtual interview sessions were audio recorded and transcribed by a professional transcription service. Participants received $50 for their participation in interviews only.

### Ethics approval and consent to participate

The University of California San Francisco Institutional Review Board reviewed this study (#19-28981) and it was determined to be exempt. All participants provided verbal informed consent to participate and all methods were performed in accordance with the relevant guidelines and regulations. The study was performed in accordance with the Declaration of Helsinki.

### Measures

The survey included demographic information, an adapted version of the everyday medical discrimination scale [28] reported in a previous publication [17], and open-ended questions. The open-ended questions included experience working in the NICU, specifically regarding healthcare team dynamics including respect and communication, and witnessed racialized care including discrimination or privileged care experienced by families. The interview guide assessed experiences with both racism and racial inequities in neonatal outcomes, and key drivers of both, including individual, institutional, and structural drivers. The interview guide also probed participants for recommendations for supporting racially marginalized families and staff.

### Data analysis

The analysis was conducted by two members of the research team who independently coded the transcripts and survey responses using a preliminary code book that included possible and anticipated themes conceptualized during interview guide development using spreadsheets (Excel Version 16.83). Additional inductive codes were added during the data analysis as previously unanticipated concepts emerged. The coding team then compared and resolved coding differences, and analyzed the data using thematic analysis. Themes were generated and refined through discussion, any disagreements on themes were resolved over several sessions while revisiting the original data and code excerpts iteratively, and consensus on themes was reached with the study team. Descriptive statistics were used to describe demographic data.

## Results

### Quantitative results

A total of 72 NICU staff members participated in study, by either completing the open-ended survey (*n* = 62, 86.1%) or additionally completing a semi-structured interview (*n* = 10, 13.9%) (see Table 1). The majority of the participants were 25–34 years old (*n* = 31, *n* = 43.1%) and identified as female (*n* = 59, 81.9%). Most participants identified racially as White (*n* = 33, 45.8%) or Asian (*n* = 16, 22.2%). NICU staff also identified as Multi-Racial (*n* = 5, 6.9%), Black (*n* = 10, 13.9%), Hispanic/Latinx (*n* = 6, 8.3%), and Other Race (*n* = 2, 2.8%). Most participating staff were employed as nurses (*n* = 33, 45.8%), followed by resident or fellow physician trainees (*n* = 13, 18.1%), attending physicians (*n* = 9, 12.5%), nurse practitioners or hospitalists (*n* = 8, 11.1%), respiratory therapists (*n* = 4, 5.6%), and other staff (*n* = 5, 6.9%). Interviews ranged in duration between 25 to 90 min, and averaged 38 min. Five main themes were generated (Table 2).

**Table 1:** Staff characteristics.

	*n*	%
Age		
25–34 yrs	31	43.1
35–44 yrs	19	26.4
45+ yrs	22	30.6
Gender		
Female	59	81.9
Male	13	18.1
Race/ethnicity		
Hispanic/Latinx	6	8.3
Black	10	13.9
White	33	45.8
Asian	16	22.2
Native Hawaiian and Pacific Islander	1	1.4
Multiracial (>1 race)	5	6.9
Middle Eastern/North African	1	1.4
Role		
Nurse	33	45.8
Trainee (resident or fellow)	13	18.1
Nurse practitioner or hospitalist	8	11.1
Attending	9	12.5
Respiratory therapist	4	5.6
Other staff	5	6.9
Response type		
Survey only	62	86.1
Survey and interview	10	13.9
Total	72	100

**Table 2:** Themes and staff interview and survey exemplar quotes.

Theme	Exemplar quotes
1 - Denial and recognition of racism	“Overall, the ICN is the least biased environment I encounter on a day-to-day basis”.
	“I don’t feel personally like I give any different care. So I’ve heard those results before and they’re very upsetting to me because I know the care that I provide”.
	“In ICN I think that racism is just there in how we treat our Black families through not necessarily giving them, not intentionally giving them worse care but I feel like we give our white families more privileged care. We don’t go as much out of our way to take care of families that aren’t like ourselves”.
2- Culture and relationships: micro/ macroaggressions	“I’ve heard people say they should go back to their country they are using up our resources; referring to Latinos”.
	“So we had a family member or parent, father, who had a swastika on his arm, you know swastika from the Nazis and a Confederate flag. […] So those sorts of things happen and those sort of microaggressions or just things like that happen and I just got to keep doing my work and show up and the swastika confederate flag and things like that and say, ‘Gosh, your baby is doing well, is there anything else I can do to help?’ you know, because that’s my job and that’s what I do. But it doesn’t make it easy”.
2 - Culture and relationships: intersectional oppression	“I’ve definitely felt the, I don’t know, just that like I have to prove myself a little bit more sometimes”.
	”[…] because of the combination of like female and minority, that adds up the layer of like their voice is not valuable because other people see that they are not capable of doing [things]”.
	“I have family members question my years of experience often or react poorly when I introduce teaching that might be ‘another way’ of doing something someone else has taught”.
3 - Lack of workforce diversity: representation	”[There is a] significant challenge in people understanding cultural context, manners of communication and it translates either in willingness or persistence with utilization of interpretation, the ways in which people give people the benefit of the doubt, and the language in which people use to describe families”.
	“I notice that when I take care of Black families they lay everything out on the table to me, their woes, and their relief that I am there”.
	“[Black nurses] tell [Black families], ‘You have to call every day and if you don’t call every day the other nurses are going to say that you don’t care.’”
3 - Lack of workforce diversity: minority tax	“I put in probably a quarter of an FTE, which is more than a day a week. Let’s just put it that way, I put in more than a day a week on diverse things and it’s all uncompensated. And I’m not the only one that does that”.
	“It was really challenging as a woman of color to constantly be someone who would challenge or offer a different perspective or encourage the medical team to think about things in a different way”.
4 - Language barriers and biased communication: language barriers	“Some of the staff was bringing Google translators to Spanish-speaking families and using that to talk to them and then I kind of saw it happen firsthand and I was like oh, this is really more of a prevalent thing than I thought. […] when I started taking care of that family there was like a lot of misinformation and they weren’t sure and none of this was their fault, I think it was the fact that we hadn’t as a healthcare system in that moment done what we needed to do for this family”.
	“[if] no one has told you how to call in to the nursery with an interpreter or how to ask for an interpreter, you may go days without knowing 1) how your kid’s doing and 2) what are things that you could be doing to try to understand your kid’s clinical condition and try to help”.
	“[Having dedicated in-person interpreters] would not only help families, because families see somebody that they can trust and they have developed a relationship with, but additionally, I think that interpreters themselves will understand the science better and the medicine better to explain it more easily and in a way that the families can understand it”.
4 - Language barriers and biased communication: biased provider communication	“Families that were white would be described as like lovely couples and that families of color, it often would be described what the family structure was”.
	“It’s like all the little markers of how people will talk about a Black family without saying that they’re Black”.
	“[…] it’s like [Black families] have a scarlet letter on [their] door”.
5 - Resource allocation	“[A white mother] was not particularly well behaved on the unit. Specifically, she was rude to staff. Unlike folks of other skin colors, there were never conversations that I’m aware of that asked about whether she could be present or needed a behavioral contract, etc”.
	“[White families were more likely to] have more primaries, receive more gifts from nurses, babies are praised as more cute etc. than our Black and Brown families”.

### Thematic results

#### Theme 1 – Denial and recognition of racism

Participants held a wide range of viewpoints on the extent to which racism was occurring within their NICU, from denial of its existence to recognition of its impact on families. The majority (*n* = 47, 89%) of survey participants acknowledged that racism exists generally within healthcare and has the potential to impact health outcomes. At times, participants extrapolated from other areas of healthcare to reason that racism may also impact care in the NICU. When probed for the etiology, several providers named unconscious bias as a leading cause of disparate care. When racism was acknowledged, many providers denied having personally experienced racist or discriminatory behavior themselves but reported witnessing other staff and families experience it in the NICU. One staff member contrasted racism with privileged care, “I think that racism is just there in how we treat our Black families through not necessarily giving them, not intentionally giving them worse care but I feel like we give our White families more privileged care. We don’t go as much out of our way to take care of families that aren’t like ourselves”. Notably, all of the participants that denied the existence of racism in the NICU, the impact of racism on health outcomes, or how racism could impact the care they provided identified as non-Hispanic White except one person who identified as Hispanic White. This sentiment was described by a staff member, “I don’t feel personally like I give any different care. So I’ve heard those results before and they’re very upsetting to me because I know the care that I provide”.

For those who acknowledged the existence of racism within their NICU, the solutions proposed to address the denial of the impact of racism on care included further educational opportunities that address unconscious bias and delineating the difference between racism versus race as a source of health disparities. A staff member identified core areas for growth of humility and self-reflection, “I think a lot of staff are not self aware. They recognize mistreatment, and they don’t like it when it happens to people they care about. Yet, they don’t see that they are sometimes perpetrators of the very behavior they claim to oppose”.

#### Theme 2 – Culture and relationships

##### Positive workplace environment

A majority (*n* = 46, 84%) of NICU staff members described their overall experience working in the NICU in a positive manner. They mentioned high job satisfaction, happiness, and great collaboration amongst providers. The NICU environment was described as welcoming, respectful, and supportive. Many providers noted the recent advances in diversity, equity, inclusion (DEI) initiatives, several citing a nursing-led initiative that aims to hold forums to identify how staff and patients experience racism, conduct educational sessions for staff, and create institutional change to address racism as contributing to this welcoming environment. A few staff members noted progress measured by the increase in staff racial diversity since the 1990s, while also highlighting that there is still a need for staff to more closely reflect the population the NICU serves. Most providers qualified their overall positive experiences and unit progress with reported observations or personal experiences of discrimination and thus a need for further improvement. One staff member summarized the sentiment of acknowledging shortcomings, remaining hopeful, and moving forward, “Bring it to the forefront so everyone knows and can make positive changes”. Another staff member offered one contributing factor for the dichotomy, “The NICU can be an intense place where people are stressed and sometimes stretched beyond their limits. This can result in curt interactions and inefficient listening/collaboration. At the same time, there can be a lot of love and comradery in the unit”.

##### Microaggressions

Racial microaggressions are “brief and commonplace daily verbal, behavioral, or environmental indignities, whether intentional or unintentional, that communicate hostile, derogatory, or negative racial slights and insults toward people of color” [29]. Micro- refers to the interpersonal level of the interaction, rather than the severity or impact of the aggression [29]. NICU staff described microaggressions that were bidirectional in nature, both directed toward staff members and patient families that originated from both staff and patient families. Several racially marginalized staff members noted experiencing a variety of microaggressions, from being confused for other staff of color to frequently being talked over during rounds (which was attributed to racism). A Black physician described an instance of stereotyping where they entered a patient room and the parent greeted them with “Oh, you must be here to fix the plumbing. We had a problem with the sink,” and racially offensive messaging from parents, including a parent wearing a “Black Labs Matter” t-shirt in the hospital. Less frequent, but still present were instances of overt interpersonal racism.

##### Intersectional oppression

Several staff members described how living at the intersection of multiple forms of oppression shaped their experiences. Racially minoritized providers who identified as female described experiences of sexism in addition to racism. One staff member reflected on the positionality of his colleagues “because of the combination of like female and minority, that adds up the layer of like their voice is not valuable because other people see that they are not capable of doing [things]”. Providers also reported differences in treatment from their colleagues and parents secondary to their age, level of experience, or their profession.

Study participants suggested promoting an environment where open conversation and feedback is encouraged and expected to address the microaggressions, in addition to bystander training to increase staff comfort with speaking up when they witness discrimination.

#### Theme 3 – Lack of workforce diversity

##### Representation

Many providers discussed the imbalance of racial composition between those providing care and those receiving care in the NICU. Black and Hispanic providers were interpreted as most underrepresented, in line with the demographic make-up of the unit that has less than national representation of Black and Hispanic staff. One staff member hypothesized that White staff may lack comfort in relating to families of color, noting, “It’s more challenging for people to, like, empathize and serve a population that they don’t see themselves in”. Conversely, staff of color described a greater ease of building relationships with racially concordant families. Occasionally, racially marginalized staff felt it necessary to coach families of color on how to avoid receiving discriminatory care. One stark example was a staff member reflecting on staff coaching parents: “[Black nurses] tell [Black families], ‘You have to call every day and if you don’t call every day the other nurses are going to say that you don’t care”.

To address the lack of racial diversity amongst staff, participants requested “equitable hiring first and foremost,” specifically more Black/Hispanic people in compensated staff and leadership roles. One Black staff member reflected on a conversation with a Black mother, ‘She said, she almost never saw anybody Black or Brown except for housekeeping. Although I took care of her baby, that was months into the hospitalization. There were just so very few Black and Brown providers. Are there some? Yes, there are some but there are very little”.

##### Minority tax

Racially marginalized providers noted feeling several types of minority tax that led to fatigue. Cultural tax, or minority tax, is the uncompensated obligation placed on minoritized people working in academic medicine [30]. One example was a disproportionate need to play a role as patient advocates. In some cases, minority tax was compounded by the sense of disempowerment of residents and fellows. Trainees comprised the majority of the staff of color participating in the study. Such trainee staff members are optimally placed to advocate for their patients due to their clinical responsibilities requiring more time at bedside, contributing to the minority tax. Staff also described their commitment to important yet uncompensated work to advance diversity, equity, and inclusion. “I put in probably a quarter of an FTE, which is more than a day a week. Let’s just put it that way, I put in more than a day a week on diverse things and it’s all uncompensated. And I’m not the only one that does that”.

The short-term solution proposed to address inadequacies of diversity in the workplace was to increase cultural humility amongst staff, enabling them to form deeper, more humanistic relationships with their patients. The proposed long-term solution was to facilitate the recruitment and retention of staff of color.

#### Theme 4 – Language barriers and biased communication

##### Language barriers

Discordant staff and parent preferred language was identified as a significant barrier to equitable care by staff. Specifically noting that non-English speaking families received unequal care that manifested mainly as less frequent parental updates, one staff member humbly admitted “I myself have avoided giving a family an update in some situations because it takes extra time to get an interpreter etc. when I would have given the same family an update had they spoken English”. Staff members also highlighted structural barriers to the use of interpreters, including time restrictions, competing duties, lack of access to in-person (gold standard) interpreters, and practical inconveniences which led to staff resorting to communicating insufficiently in the family’s preferred language such as using online translation services applications such as Google Translate rather than a certified medical interpreter.

The solution most frequently proposed to address this issue was making timely, consistent interpretation with an approved interpreter a “cultural norm” and having a dedicated team of in-person interpreters. In addition, diversification of the workforce with more staff who speak languages other than English, described in the previous theme.

##### Biased provider communication

The content of conversations had by staff was noted by several participants as contributory to racist narratives. Other staff mentioned the labeling of families as “difficult” or “aggressive” which led to distancing of staff from the labeled families, resulting in superficial relationships and less frequent updates. Some families that could not be at their infant’s bedside as frequently as others were labeled as “uninvolved” and this resulted in their parenting capacity being questioned despite evidence to the contrary. These negative narratives, though they start on an individual, interpersonal level, become enmeshed into the dominant narrative surrounding the family during their stay in the NICU.

Proposed solutions to address racist narratives included staff “getting to know our patients and their families on a community level” by spending time with communities of color in their spaces either socially or through volunteering, more frequent DEI-focused training sessions, and training in cultural competency.

#### Theme 5 – Resource allocation

Tangible disparities in the distribution of valued institutional resources were noted by many staff. Families that appeared to be from higher socio-economic backgrounds and tended to be of a White racial background, were more likely to receive exceptions for providing donor breast milk to their infant, desirable single room assignments, and longer interactions with attending physicians, while “[racially marginalized families received] noticeably less attention from the medical team including attending physicians and nurse managers”. Some staff members noted more tolerance for typically unacceptable behaviors and more laxity with unit rules for white families: “[A White mother] was not particularly ‘well behaved’ on the unit. Specifically, she was rude to staff. Unlike folks of other skin colors, there were never conversations that I’m aware of that asked about whether she could be present or needed a behavioral contract, etc”.

The most frequently proposed solution to address inequitable resource allocation was to encourage self-reflection and introspection by “continu[ing] open dialogue and iterative DEI training. A commitment to being anti-racist is one that requires ongoing work and redoubling of efforts”. Specifically, participants requested de-escalation training, trauma-informed care training, and multidisciplinary training about health disparities and cultural competency. In terms of resource allocation, staff called for equitable primary nurse and room allocation. Finally, resources dedicated to detecting and addressing racial inequities on a national, regulatory level were called for by a staff member, “I think if we treated disparate care due to race as seriously as we treated bloodstream infections and accidental extubation, those are things that we look at every single day, that would make a huge difference”.

## Discussion

This study provides staff perspectives on witnessing and experiencing various forms of racism in the NICU setting, while also highlighting local workplace strengths and overall progress. This study widens the focus from the impact of racism on parents and families to also considering staff experiences of both witnessing and being subject to structural racism and thus provides a unique opportunity to obtain a more comprehensive assessment of the impact of racism in this setting.

There are several similarities between the present analysis of staff experiences and a previous analysis of parent experiences within the REJOICE study [31]. In both analyses, there were stark differences of experiences that range from positive to negative that occurred both within and between individuals [29]. The coexistence of care and harm described by parents, and positive and negative workplace environments described by staff members, are evidence of the multitude and complexity of interactions between individuals [31]. Each person has many experiences within a space, and groups of individuals can experience the space differently. For example, one parent can describe both positive and negative staff interactions [31], and racially marginalized staff members are at higher risk of recognizing and experiencing racism and can have positive experiences in the workplace [17]. Both analyses and Sigurdson et al. previously reported experiencing the effects of racism at the interpersonal and institutional levels [32]. Other qualitative studies further corroborate our witnessed reports of racism and discrimination toward patients, spanning countries and medical disciplines [14, 15]. Specifically, language barriers and biased communication were also themes from a recent study interviewing caregivers and staff in a recent qualitative study in a southeastern NICU [33].

There were also nuanced differences between the REJOICE parent and staff perspectives described in these qualitative analyses, supported by the significant quantitative differences in reporting racism [17]. In addition to staff echoing concerns about parent experiences, racially marginalized staff in the NICU highlighted their own experiences with interpersonal and institutional racism. Staff members reported minority tax, advocacy fatigue, and subjection to microaggressions, racism, and discrimination from both parents and colleagues. These findings are aligned with previous literature of workplace experiences of racism [14, 26, 27, 32]. In summary, racism does not differentiate based on patient or staff status for both the perpetrating and receiving role, reflecting the infiltrative nature of racism.

Institutional strengths identified by staff members included improved awareness of racial inequities, provider diversity, newly dedicated forums to advancing DEI and antiracism, and a willingness to have a growth mindset to acknowledge opportunities for improvement across unit staff. Many participants recommended DEI training as a solution. There are studies that have found positive change in knowledge, attitudes or awareness after training, but few studies on DEI training have measured participant skills or behavior change [34]. The evidence-base is further compromised by the current heterogeneous nature of DEI training interventions [34]. Therefore DEI training is one potentially impactful interpersonal-level intervention that should be considered, but only in addition to other policy, organizational, and community-level interventions [35]. Staff recommendations also included support for perpetual education with practical application, open dialogue, and increasing workforce diversity, rather than a single mandatory DEI didactic training. These endeavors are all dependent on staff recognition that bias, racism, and discrimination can impact healthcare [14]. In our study, the spectrum to which bias in healthcare is recognized is varied, highlighting the importance of education and combating racism [14, 16, 36, 37]. Future research is needed for evidence-based programs that encourage the self-reflection needed to acknowledge and confront personal biases to create lasting change [38–41].

At an institutional level, participants recommended that efforts to recruit and retain staff members of color increase, hearkening to the American Academy of Pediatrics’ long-standing call to increase the number of providers that are underrepresented in medicine [10, 14, 16, 42, 43]. Unfortunately, the racial and ethnic composition of physician and nursing workforce in neonatology is either stagnant or becoming more homogenous, demonstrating the importance of diversifying staff in neonatology [10–13, 43]. Racially minoritized faculty in particular, are underrepresented in both nursing and physician fields [10, 11, 13, 44]. The downstream impacts of lack of diversity and resultant minority tax are consequential and varied, impacting the future generation of healthcare providers, current work environment, and patients’ experiences and outcomes [45–47]. Aligned with Sigurdson et al., to make care more equitable at the institutional level, dedicated full-time in-person Spanish interpreters and more evenly distributed room and primary nurse allocation procedures were also recommended [18, 43].

All of the recommendations provided are achievable solutions to advance racial equity and in combination with the growth-mindset of staff members are foundations for positive change. Noticing and naming racism is the first barrier to combating racism [48]. Unfortunately, common sociocultural norms for denying racism exist [49]. Many NICUs do not yet routinely screen for staff or caregiver experiences with racism nor monitor for evidence of racial inequities. Without acknowledging the existence of racism, healthcare organizations including NICUs cannot make positive changes to address it.

The findings of our study should be interpreted within the context of the study’s design. This study was conducted at a single center level IV NICU, potentially limiting the generalizability of our findings to other centers. Additionally, not all staff members participated in the study, which could lead to a selection bias with respondents more likely to have extreme or polarizing views. When using virtual interview methodology, developing a rapport and reading body language may limit the full breadth of interpersonal communication and interpretation for sensitive topics. The study was also conducted in part during the COVID-19 pandemic and the racial justice movement following the murder of George Floyd, which likely exacerbated existing barriers to care, increased staff burnout levels, and increased overall awareness and likely impacted responses to racial injustice.

## Conclusion

Racial inequities in healthcare continue to exist across healthcare disciplines, and the NICU is no exception [1, 6, 8]. Our study explores the ways in which NICU staff members experience structural racism through open-ended surveys and interviews. We found several themes including a spectrum of awareness of racial inequities, facilitating and barriers to a protective culture and relationships, inadequate workplace diversity, language and communication challenges, and inequitable resource allocation. Several potential solutions were proposed by staff members, and these, in combination with the NICU’s growth mindset, highlights a path forward to improving racial health disparities. Future research should be directed to evaluation and implementation of these recommendations.

## Supplementary information

Supplemental Table 1

## Data Availability

The datasets generated during and analyzed during the current study are not publicly available due to concerns that even when de-identified, there is significant risk of identifying participants and their responses, given the small sample size in a small patient and workplace community. Excerpts are available from the corresponding author on reasonable request.
